# The R2R3 MYB Transcription Factor *MYB71* Regulates Abscisic Acid Response in *Arabidopsis*

**DOI:** 10.3390/plants11101369

**Published:** 2022-05-21

**Authors:** Yuxin Cheng, Yanxing Ma, Na Zhang, Rao Lin, Yuan Yuan, Hainan Tian, Saddam Hussain, Siyu Chen, Wenting Yang, Ling Cai, Yingying Li, Xiaoping Wang, Tianya Wang, Shucai Wang

**Affiliations:** 1 Key Laboratory of Molecular Epigenetics of MOE, Northeast Normal University, Changchun 130024, China; chengyx104@nenu.edu.cn (Y.C.); mayx@cau.edu.cn (Y.M.); zhangn906@nenu.edu.cn (N.Z.); linr944@nenu.edu.cn (R.L.); yuany759@nenu.edu.cn (Y.Y.); tianhainan2012@126.com (H.T.); botanistonline@yahoo.com (S.H.); 20200049@jlbtc.edu.cn (W.Y.); c806593655@163.com (L.C.); liyy857@nenu.edu.cn (Y.L.); wangty309@nenu.edu.cn (T.W.); 2 Laboratory of Plant Molecular Genetics & Crop Gene Editing, School of Life Sciences, Linyi University, Linyi 276000, China; 23329915.ok@163.com (S.C.); wangxp439@nenu.edu.cn (X.W.)

**Keywords:** *MYB71*, transcription factor, abscisic acid, abiotic stress, *Arabidopsis*

## Abstract

Abscisic acid (ABA) regulates plant responses to abiotic stresses via regulating the expression of downstream genes, yet the functions of many ABA responsive genes remain unknown. We report here the characterization of *MYB71*, a R2R3 MYB transcription factor in regulating ABA responses in *Arabidopsis*. RT-PCR results show that the expression level of *MYB71* was increased in response to ABA treatment. *Arabidopsis* protoplasts transfection results show that *MYB71* was specifically localized in nucleus and it activated the *Gal4:GUS* reporter gene when recruited to the *Gal4* promoter by a fused DNA binding domain GD. Roles of *MYB71* in regulating plant response to ABA were analyzed by generating *Arabidopsis* transgenic plants overexpression *MYB71* and gene edited mutants of *MYB71*. The results show that ABA sensitivity was increased in the transgenic plants overexpression *MYB71*, but decreased in the *MYB71* mutants. By using a DEX inducible system, we further identified genes are likely regulated by *MYB71*, and found that they are enriched in biological process to environmental stimuli including abiotic stresses, suggesting that *MYB71* may regulate plant response to abiotic stresses. Taken together, our results suggest that *MYB71* is an ABA responsive gene, and *MYB71* functions as a transcription activator and it positively regulates ABA response in *Arabidopsis*.

## 1. Introduction

MYB transcription factors are one of the largest transcription factor families in plants, and there are ~200 genes in *Arabidopsis* encoding MYB transcription factors [1]. According to the number of N-terminal DNA binding domain repeats, MYB family transcription factors can be divided into four subfamilies (i.e., 1R or R3 MYBs, 2R or R2R3 MYBs, 3R or R1R2R3 MYBs and 4R or R1R2R3R4 MYBs), with R2R3 MYB proteins being the largest MYB transcription factor subfamily with 126 members in *Arabidopsis* [1,2].

It has been shown that members of the R2R3 MYB transcription factors in *Arabidopsis* have several different functions. Some of the R2R3 MYB transcription factors are able to regulate plant growth and development, as examples, MYB7 and MYB70 regulate seed germination [3,4], MYB76 regulates seed fatty acids accumulation [5], ASYMMETRIC LEAVES1 (AS1) regulates leaf patterning and shoot morphogenesis [6], MYB2 regulates axillary meristem formation [7], MYB21 and MYB24 regulate anther development [8,9], AtMYB77 regulates lateral root formation [10], whereas GLABRA1 (GL1) and WEREWOLF (WER) regulates trichome and root hair formation, respectively [11,12]. Some of the R2R3 MYB transcription factors regulate secondary metabolisms, for example, MYB94 regulates cuticular wax biosynthesis [13], AtMYB15, AtMYB46, AtMYB52, AtMYB54, AtMYB58, AtMYB63, AtMYB85 and AtMYB103 regulate secondary cell wall biosynthesis [14,15,16,17], MYB115 regulates proanthocyanidin biosynthesis [18], PRODUCTION OF ANTHOCYANIN PIGMENT 1 (PAP1), PAP2, MYB113 and MYB114 regulate anthocyanin biosynthesis [19,20], and MYB4 regulates anthocyanin and proanthocyanidin biosynthesis [21]. Some of the R2R3 MYB transcription factors are involved in the regulation of plant response to abiotic stresses. For instance, MYB32 regulates drought tolerance [22], MYB20, MYB25, MYB30 and MYB49 regulates salt tolerance [23,24,25,26], MYB68 regulates heat and drought tolerance [27], and MYB109 regulates osmotic stress response [28]. Yet the functions of a large portion of the R2R3 MYB transcription factors remained uncharacterized.

As the key stress hormone, ABA (abscisic acid) regulates the expression of ABA responsive genes via signal transduction and thereby plant responses to abiotic stresses including drought, salinity, cold and heat [29,30,31,32,33,34,35,36]. Whereas ABA signaling is medicated by several different regulators including the receptor proteins PYR1/PYL/RCAR (Pyrabactin resistance 1/PYR1-like/Regulatory component of ABA receptor), the negative regulators A-group PP2Cs (PROTEIN PHOSPHATASE 2C) phosphatases, the positive regulators SnRKs (NONFERMENTING 1 (SNF1)-RELATED PROTEIN KINASES) kinases, and the downstream ABF/AREB/ABI5-type bZIP (basic region leucine zipper) transcription factors [29,31,34,35,36,37,38,39]. Consistent with the roles of ABA in regulating plant responses to abiotic stresses, mutation and/or overexpression of the ABA signaling regulator genes affected plant tolerance to abiotic stresses. In most of the cases, overexpression of the regulator genes such as the PYR1/PYL/RCAR receptor genes led to enhanced, whereas loss-of-function of the regulator genes including the PYR1/PYL/RCAR receptor genes, the *SnRK2s* genes, and the ABF/AREB/ABI5 transcription factor genes led to reduced abiotic stress tolerance in plants [40,41,42,43].

As mentioned above, ABA regulates the expression of ABA responsive genes and thereby plant responses to abiotic stresses, suggest that ABA response genes are involved in the regulation of plant responses to abiotic stresses. Indeed, some ABA response genes have been shown to be involved in the regulation of plant abiotic stresses responses. For example, the expression level of the R2R3 MYB transcription gene *MYB44* was increased in response to ABA treatment, and *Arabidopsis* transgenic plants overexpressing *MYB44* showed an enhanced tolerance to drought and salt stresses [44], the heat shock factor gene *HSFA6b* is an ABA responsive gene, and it plays a role in regulating drought and salt tolerance [45], overexpression of *AFBA1* (*ABA-responsive FBA (F-box-associated) domain-containing protein 1*) enhanced plant drought [46], and the expression of bHLH transcription factor gene *bHLH112* is induced by ABA, and it positively regulate drought and salt tolerance in *Arabidopsis* [47]. Yet the functions of most ABA responsive genes remain to be characterized.

By analyzing uncharacterized ABA responsive genes, and examining their roles in regulating ABA responses first, we have successful identified several abiotic stress response regulator in *Arabidopsis*. For example, we found that the expression of two closely WD40 genes, *ABA induced WD40-repeat 1* (*AIW1*) and *AIW2*, the non-DNA binding bHLH transcription factor gene *Paclobutrazol Resistance 6* (*PRE6*), and a novel transcription factor family genes *ABA-induced transcription repressors* (*AITRs*) were all induced by ABA treatment [35,48,49], and then we showed that the *aiw1 aiw2* mutants and the transgenic *Arabidopsis* plants overexpressing *PRE6* showed increased sensitivity to ABA [48,49], whereas loss-of-function of *AITR* genes in *Arabidopsis* led to reduced sensitivity to ABA [35,36], suggest that these regulators regulate ABA response in *Arabidopsis*, indicating that they may be involved in the regulation of plant responses to abiotic stresses. Indeed, salt tolerance was reduced in *Arabidopsis* transgenic plants overexpressing *AIW1* [48], *Arabidopsis* transgenic plants overexpressing *PRE6* showed enhanced salt tolerance [49], and loss-of-function of *AITR* genes led to enhanced tolerance to abiotic stresses including drought as well as salt [35,36]. These results indicate that it is practicable to identify abiotic stress regulators by analyzing the roles of uncharacterized ABA responsive genes in regulating ABA responses in plants.

We report here the characterization of *MYB71*, a R2R3 MYB transcription factor in regulating ABA response in *Arabidopsis*. We found that the expression level of *MYB71* is increased in response to ABA, *MYB71* is able to activate reporter gene expression, and overexpression or mutation of *MYB71* affected plant response to ABA.

## 2. Results

### 2.1. Expression of MYB71 Is Induced by ABA Treatment

**Figure 1:**
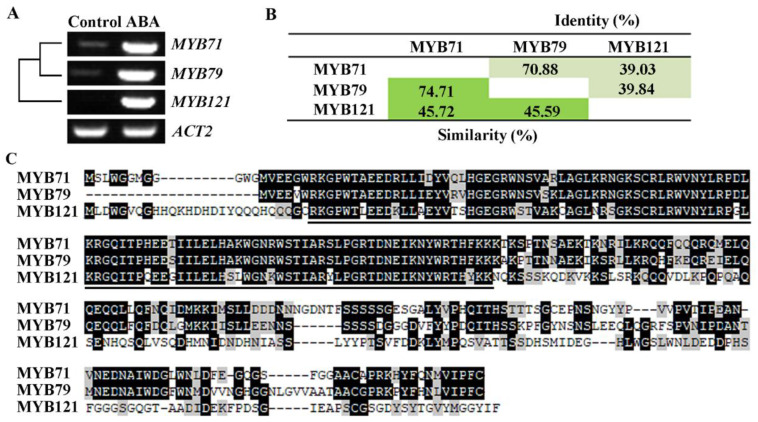
*MYB71* and its closely related genes *MYB79* and *MYB121* are ABA responsive genes. (**A**) Expression of *MYB71*, *MYB79* and *MYB121* in response to ABA. Ten-day-old Col wild type seedlings were mock treated or treated with 50 µM ABA for 4 h, total RNA was isolated, and cDNA was synthesized and used for RT-PCR analysis. *ACT2* was used as a control. (**B**) Percentage of amino acid identity and similarity of *MYB71*, *MYB79* and *MYB121*. Full-length amino acid sequences of *MYB71*, *MYB79*, and *MYB121* were subjected to SIAS (http://imed.med.ucm.es/Tools/sias.html, accessed on 25 May 2018) for amino acid identity and similarity assay. (**C**) Amino acid sequence alignment of *MYB71*, *MYB79*, and *MYB121*. Identical amino acids were shaded in dark, and similar ones in gray. Underlines indicate the R2R3 MYB repeats.

In the process of identifying abiotic stress regulators by examining the roles ABA response genes in regulating plant ABA responses as described previously [35], we found that the expression of the *Arabidopsis* R2R3 MYB transcription factor gene *MYB71* was induced by ABA treatment (Figure 1A). Since *MYB71* is closely related to *MYB79* and they formed a function uncharacterized small cluster together with *MYB121* [2], we also examined the expression of *MYB79* and *MYB121* in response to ABA. As shown in Figure 1A, the expression levels of *MYB79* and *MYB121* increased sharply after the seedlings were treated with ABA.

Amino acid sequence identify and similarity assays indicates that *MYB71* shared 70.88% amino acid identify and 74.71% amino acid similarity, respectively with *MYB79*, by only 39.03% amino acid identify and amino acid similarity 45.72%, respectively, with *MYB121* (Figure 1B). Whereas amino acid sequence alignment shows that all of the three MYB proteins have a highly conserved R2R3 domain at theirs N-terminus, but a C-terminus with less amino acids conserved (Figure 1C).

### 2.2. MYB71 Functions as a Transcription Activator

Considering that transcription factors may have transcriptional activation or repression functions, and should be functioned in nucleus, we therefore examined the subcellular localization and transcription activities of *MYB71* by using *Arabidopsis* protoplast transient transfection assays.

**Figure 2:**
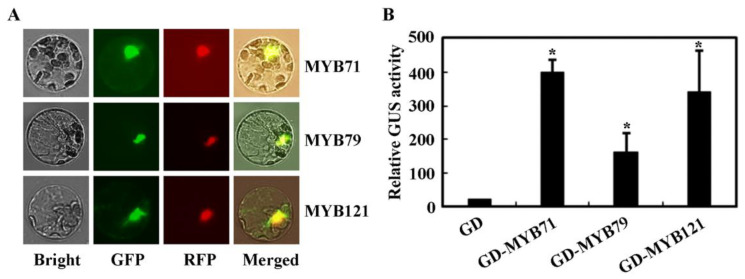
*MYB71* and its closely related proteins *MYB79* and *MYB121* are transcription activators. (**A**) Subcellular localization of *MYB71*, *MYB79*, and *MYB121*. Plasmids of the effector genes *GFP-MYB71*, *GFP-MYB79*, and *GFP-MYB121* were co-transfected, respectively, with the nuclear marker gene *NLS-RFP* into *Arabidopsis* protoplasts. The transfected protoplasts were incubated in dark for 18–20 h at room temperature, then GFP and RFP fluorescence was observed under a confocal microscope. (**B**) Transcriptional activities of *MYB71*, *MYB79* and *MYB121*. Plasmids of the effector genes *GD*, *GD-MYB71*, *GD-MYB79*, *GD-MYB121* were co-transfected, respectively with the reporter gene *Gal4:GUS* into protoplasts. The transfected protoplasts were incubated in dark for 20–22 h at room temperature, the GUS activities were measured by using a microplate reader. Data represent the mean ± SD of three replicates. * Significantly different from the control (*p* < 0.05).

To examine the subcellular localization of *MYB71*, plasmids of *MYB71-GFP* and the nucleus indicator gene *NLS-RFP* were co-transfected into *Arabidopsis* protoplasts, and the GFP and RFP fluorescence was observed under a cofocal microscope. As shown in Figure 2A, *MYB71* was predominantly localized in the nucleus. Since *MYB71*, *MYB79* and *MYB121* formed a small cluster [2], we also examined the subcellular localization of *MYB79* and *MYB121*, and found that both of them were also predominantly localized in the nucleus (Figure 2A).

For *MYB71* transcription activity examination, plasmids of the reporter gene *Gal4:GUS* and the effect gene *GD-MYB71* or the control gene *GD* were co-transfected into *Arabidopsis* protoplasts, and GUS activities were measured by using a microplate reader. In this system, *MYB71* is able to bind to the *Gal4* promoter in the reporter gene via the fused GD domain. As shown in Figure 2B, expression of the reporter gene was activated by transfection of effector gene *GD-MYB71*, indicating that *MYB71* function as a transcription activator. Similar, we also found that *MYB79* and *MYB121* function as transcription activators (Figure 2B).

### 2.3. Generation of the 35S:MYB71 Transgenic Plants and the MYB71 Mutants

**Figure 3:**
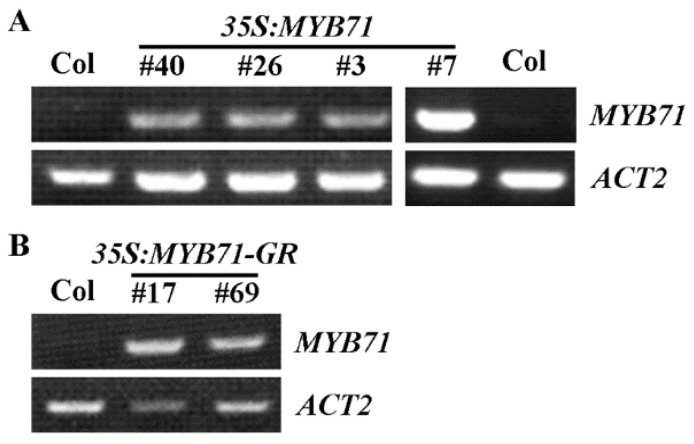
Expression of *MYB71* in the *35S:MYB71* and *35S:MYB71-GR* transgenic plants. (**A**) Expression of *MYB71* in the *35S:MYB71* transgenic plants. (**B**) Expression of *MYB71* in the *35S:MYB71-GR* transgenic plants. Total RNA was isolated from 10-day-old homozygous transgenic plants and RT-PCR was used to examine the expression of *MYB71*. Expression of *ACT2* was used as a control.

In order to study the roles of *MYB71* in regulating ABA responses in *Arabidopsis*, we generated transgenic overexpression plants and gene edited mutants for *MYB71*. Transgenic plants overexpressing *MYB71* were generated by transforming the Col wild type plants with the *35S:MYB71* construct, and selecting homozygous transgenic plants in the T3 generation. Four different lines of homozygous transgenic plants were obtained, and RT-PCR results show that the expression level of *MYB71* was increased in all of the four lines when compared with that in the Col wild type plants, with line #7 has the highest expression level (Figure 3A). Transgenic plants overexpressing *MYB71-GR* fusion gene was also generated in order to identify genes that may be regulated by *MYB71* (Figure 3B).

**Figure 4:**
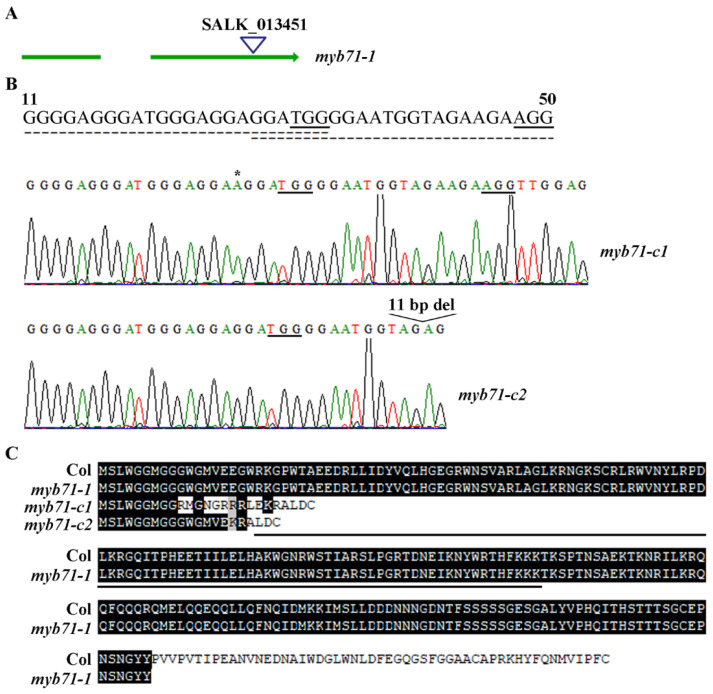
Isolation of T-DNA insertion mutant and generation of gene edited mutants of *MYB71*. (**A**) T-DNA insertion position of the *myb71-1* single mutant. Seeds of the SALK_013451 T-DNA insertion line were obtained from ABRC, and PCR was used to identify homozygous mutant. (**B**) Gene editing status of *MYB71* in the *myb71-c1* and *myb71-c2* mutants. DNA was isolated from leaves of transgene-free T2 plants, and PCR was used to amplify the genome sequence of *MYB71*. The PCR products were recovered and sequenced, and sequencing results were compared with genome sequence of *MYB71* to check the editing status. Top panel shows the two overlapped target sequences. Dash lines indicate the target sequences, solid lines indicate the PAM sites, and star indicated the single nucleotide inserted. (**C**) Alignment of *MYB71* amino acid sequence in the Col wild type and the *MYB71* mutants. The open-reading frame (ORF) of *MYB71* sequence in the *myb71-c1* and *myb71-c2* single mutants were identified by using ORFfinder (http://www.ncbi.nlm.nih.gov/orffinder/, accessed on 31 May 2019), and corresponding amino acid sequences were used for alignment with the amino acid sequence of *MYB71*. The amino acid sequence for *MYB71* in the *myb71-1* mutant corresponding to the amino acids encode by the CDS before the T-DNA insertion. Underlines indicate the R2R3 MYB repeat.

There is one T-DNA insertion line for *MYB71* available from TAIR, we therefore isolated the single mutant *myb71-1* from the T-DNA insertion line (Figure 4A).

Since there is only one T-DNA insertion mutant, we decided to generate gene edited mutants for *MYB71* by using CRISPR/Cas9 gene editing, a method that can be used to generate transgene-free mutants [50,51,52,53]. Two overlapped target sequences near the start the condon of *MYB71* was selected (Figure 4B), and gene editing construct was generated and used for plant transformation. By sequencing the *MYB71* gene in the transgenic plants, and isolated transgene-free mutants based on the PCR amplification of Cas9 vector fragment, we successfully obtained two transgene-free *MYB71* mutants (i.e., *myb71-c1* and *myb71-c2*).

In the *myb71-c1* mutant, a single nucleotide insertion was occurred in the first target sequence, whereas in the *myb71-c2* mutant, an 11 bp deletion was occurred in the second target sequence (Figure 4B). Both the single nucleotide insertion and the 11 bp deletion in *MYB71* gene resulted in a few amino acid substitutions and premature stops for *MYB71* (Figure 4C). The predicated proteins encoded by the mutated *MYB71* gene in both the *my71-c1* and *myb71-c2* mutants contain only ~20 amino acids before the R2R3 domain in *MYB71*, whereas the predicated truncated *MYB71* protein in the T-DNA insertion mutant *myb71-1* contains the entire R2R3 domain and part of the C-terminal domain (Figure 4C).

### 2.4. The 35S:MYB71 Transgenic Plants Are Hypersensitivity, Whereas the MYB71 Mutants Are Hyposensitive to ABA

By using the transgenic overexpression plants and gene edited mutants of *MYB71* generated, we examined if *MYB71* may regulate ABA response by using seed germination and cotyledon greening, assays commonly used for examining plant responses to ABA.

**Figure 5:**
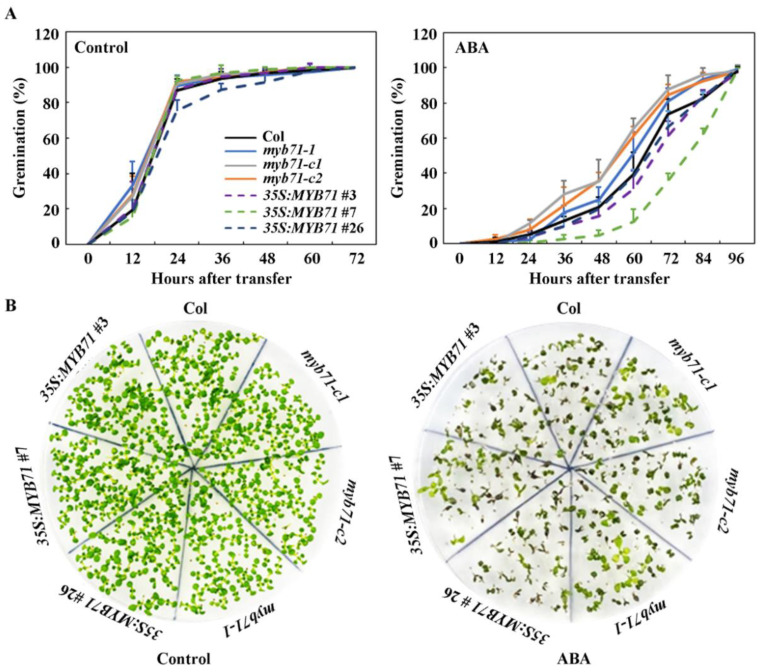
Effects of ABA on seed germination and cotyledon greening of the Col wild type, the *35S:MYB71* transgenic plants and the *MYB71* single mutants. (**A**) Effects of ABA on seed germination of the Col wild type, the *35S:MYB71* transgenic plants, and the *MYB71* single mutants. Sterilized seeds of the Col wild type, the *35S:MYB71* transgenic plants, and the *MYB71* single mutants were plated on 1/2 MS plates with or without 1 µM ABA. The plates were kept at 4 °C and in darkness for 2 days, and then transferred to a growth room. The number of seeds germination was counted at the time points indicated, and percentage of germination was calculated. Data represent the mean ± SD of three replicates. (**B**) Effects of ABA on cotyledon greening of Col wild type, the *35S:MYB71* transgenic plants, and the *MYB71* single mutants. Pictures were taken 12 days after the transfer.

Sterilized seeds of the Col wild type, the transgenic overexpression plants and gene edited mutants of *MYB71* generated were plated on 1/2 MS plates with or without 1 µM ABA, and seeds germination were counted at indicated and percentage of germination was calculated. We also included the *MYB71* T-DNA insertion mutant in our assays. As shown in Figure 5A, in the control plates, almost all of the seeds including seeds of the Col wild type, the *35S:MYB71* transgenic plants, the gene edited mutants *myb71-c1* and *myb71-c2*, and the T-DNA insertion mutant *myb71-1* germinated 36 h after the plates were transferred to a growth room. On the other hand, germination of the seeds of all of the plants was inhibited in the ABA-containing plates. However, lower germination rate was observed for the *35S:MYB71* transgenic plant seeds, whereas higher germinated rate was observed for the *MYB71* mutant seeds when compared with that of the Col wild type seeds (Figure 5A). There results suggest that the *MYB71* mutants were less sensitive but the *35S:MYB71* transgenic plants were more sensitive to ABA.

Similar, the cotyledon greening assay results also indicate that the *MYB71* mutants were less sensitive, whereas the *35S:MYB71* transgenic plants were more sensitive to ABA (Figure 5B). These results suggest that MYB71 is a positive regulator of ABA response in *Arabidopsis*.

### 2.5. MYB71 Affected Genes Are Enriched in Plant Response to Environmental Stimuli

Since *MYB71* is a transcription factor, to explore the functional mechanism of *MYB71*, we decide to identify the genes that may be regulated by *MYB71*. To do that, we generated transgenic plants overexpressing *MYB71-GR*, in which the *MYB71* protein was fused with the glucocorticoid receptor (GR). Under normal condition, the fusion proteins cannot get into the nucleus, therefore are not functional. However, when the transgenic plants were treated with dexamethasone (DEX), the fusion proteins can move into nucleus, and *MYB71* is able to regulate the expression of its downstream genes.

We therefore treated the *35S:MYB71-GR* transgenic seedlings with DEX, isolated RNA from the treated and control seedlings, and performed transcriptome analysis. Since *MYB71* functions as a transcription activator (Figure 2B), we thus focused on the genes that were upregulated in the DEX treated seedlings. We found that only 26 genes were upregulated at least two folds in the DEX treated seedlings (Table S1).

**Figure 6:**
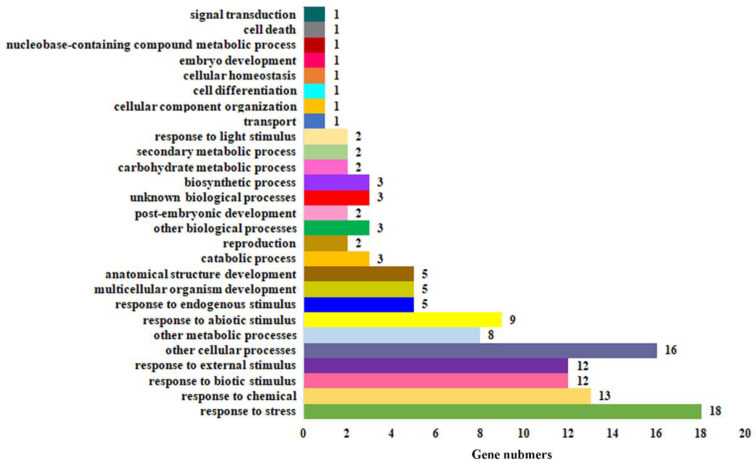
Functional categorization of the different expressed genes. The 26 genes up-regulated at least 2 folds in *35S:MYB71-GR* transgenic plant treated with DEX were used for functional categorization annotation by using TAIR gene ontology (GO) annotation (https://www.arabidopsis.org/tools/bulk/go/index.jsp, accessed on 14 September 2021). The number of genes in different biological process was indicated.

GO (Gene Ontology) analysis show that the 26 upregulated genes are involved in multiple biological processes including plant growth and development, signal transduction, and secondary metabolic (Figure 6). However, a majority of the genes are involved in biological processes related to environmental stimuli. For example, 18 genes are involved in response to stress, 13 to chemical, 12 to biotic stimulus and external stimulus, and 9 to abiotic stimulus (Figure 6).

### 2.6. Some of the Genes Regulated by MYB71 Are Related to Plant Response to Environmental Stimuli and Seed Germination

**Figure 7:**
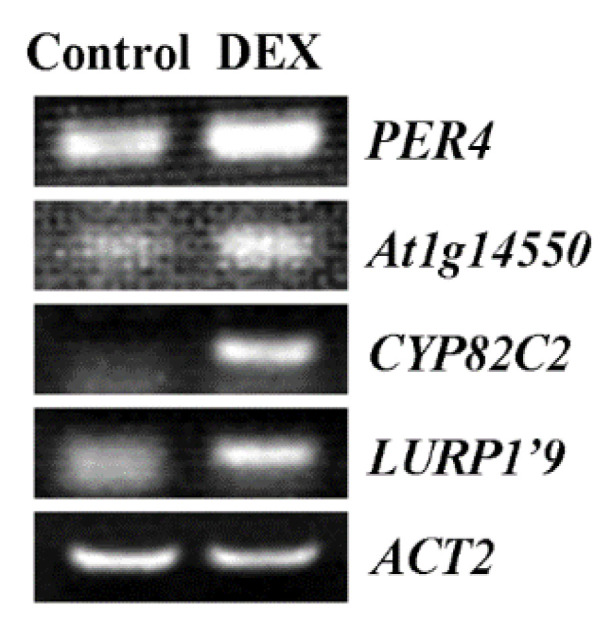
RT-PCR validation of some different expressed genes. Four genes up-regulated at least 2.0-fold in *35S:MYB71-GR* transgenic plant treated with DEX were selected and examined by RT-PCR. The expression of *ACT2* was used as a control.

Among the 26 upregulated genes in DEX treated *35S:MYB71-GR* transgenic seedlings, we found several genes have been functional characterized, for example, LURP1 and CYP82C2 are involved in pathogen defense [54,55,56], PER4 has been shown to regulated seed germination [57,58], and *At1g14550* is a tandem repeat gene of *PER4*, and encodes a protein closely related to PER4 [57]. We therefore take these genes as examples to compare their expression in DEX treated and control seedlings. As shown in Figure 7, the expression levels of all of the four genes were clearly increased in DEX treated seedlings.

## 3. Discussion

ABA is an important hormone that can trigger plants responses to various abiotic stresses, including salinity, drought, high temperature, chilling, and oxidative stress [59,60]. Since there is a close relationship between ABA and abiotic stress tolerance in plants, examining the roles of ABA response genes in regulating plant responses to ABA will help to elucidate the mechanisms of plant abiotic stress tolerance [47]. R2R3 MYB proteins are the largest subfamily of the MYB transcription factor family in *Arabidopsis* with multiple functions [1,2]. Several R2R3 MYBs such as MYB32, MYB37, MYB44, have been shown to be involved in the regulation of ABA responses [22,44,61]. In this study, we provide evidence here that *MYB71* is an ABA responsive gene, and *MYB71* positively regulates ABA response in *Arabidopsis*.

Our data show that the expression level of *MYB71* increased dramatically in response to ABA treatment, in addition, its closely related R2R3 MYB genes *MYB79* and *MYB121* are also ABA responsive genes (Figure 1). By generating *35S:MYB71* transgenic plants and gene edited *MYB71* mutants (Figure 3 and Figure 4), and examining their responses to ABA by using seed germination and seedling greening experiments, we found that ABA sensitively was increased in the *35S:MYB71* transgenic plant, but decreased in the *MYB71* mutants (Figure 5). We also noted that, among the three different lines of the *35S:MYB71* transgenic plants, highest ABA sensitivity was observed in line #7 (Figure 5), and the highest expression level of *MYB71* was also observed in line #7 (Figure 3). On the other hand, the gene edited *MYB71* mutants showed higher ABA tolerance than the T-DNA insertion mutant *myb71-1* (Figure 5). Considering that both of the gene edited *MYB71* mutants are likely loss-of-function mutants, as the predicated ORF (open reading frame) of *MYB71* in the mutants only encodes about 20 amino acids before the R2R3 MYB domain in the *MYB71* protein, whereas the T-DNA insertion mutant *myb71-1* may not be a loss-of-function as the CDS (coding sequence) before the T-DNA insertion encodes the entire R2R3 MYB domain and part of the C-terminal domain (Figure 4), there results further confirmed that *MYB71* is a positive regulator of ABA response.

It has been shown that some of the R2R3 MYB transcription factor whose gene expression can be regulated by ABA may play a feedback regulating role in ABA signaling. For example, MYB44 is able to suppress the expression of PP2C genes [44], MYB32 positively regulated the expression of *ABSCISIC ACID-INSENSITIVE 3* (*ABI3*), *ABI4* and *ABI5* [22], and MYB37 is also able to regulate the expression of some ABA signaling key regulator genes as well as some other ABA responsive genes [61]. By using a DEX inducible system, we try to identify genes that may be regulated by *MYB71*, a total of 26 genes were found to be unregulated no less than 2 folds in the DEX treated *35S:MYB71-GR* transgenic plants (Table S1). Considering that *MYB71* functions as a transcription activator (Figure 2), these genes are likely regulated by *MYB71*, and some of them may be directly targets of *MYB71*. However, none of them are ABA signaling key regulator genes, indicating that *MYB71* may not able to regulate the expression of ABA signaling key regulator genes. Therefore, it will be of interest to investigate how *MYB71* may regulate ABA response in *Arabidopsis*.

By charactering unknown function ABA responsive genes, we have successfully identified several different types of transfection factors as abiotic stress response regulators in *Arabidopsis*, including the WD40 transcription factors AIW1 and AIW2 [48], the non-DNA binding bHLH transcription factor PRE6 [49], and the novel transcription factor family AITRs [35]. Similar to these transcription factors, the expression of *MYB71* is regulated by ABA (Figure 1), and *MYB71* is involved in the regulation of ABA responses in *Arabidopsis* (Figure 5). GO analysis of the genes that may regulated by *MYB71* also show that most of the genes are involved in the process of response to environmental stimuli, including abiotic stresses, suggest that *MYB71* may be involved in the regulation of plant responses to abiotic stresses. However, further evidence is required to support a role of *MYB71* in regulating plant abiotic stress responses. 

Furthermore, as the expression of both *MYB79* and *MYB121* are also induced by ABA, *MYB71* is closely related to *MYB79*, and they shared high amino acid identity and similarity at full length amino acid level, and *MYB121* also shared high amino acid identity and similarity with *MYB71* and *MYB79* at the R2R3 repeat domain, but not the C-terminus (Figure 1). It will be of interest to examine if *MYB79* and *MYB121* are involved in regulating plant responses to ABA and abiotic stresses, and if they have similar functions as *MYB71*.

## 4. Materials and Methods

### 4.1. Plant Materials and Growth Conditions

The wild-type Columbia-0 (Col) ecotype *Arabidopsis* (*Arabidopsis thaliana*) was used for protoplasts isolation, and for plant transformed to generate transgenic plants and gene edited mutants. Seeds of the SALK_013451 T-DNA insertion line in Col background were obtained from ABRC (*Arabidopsis* Biological Resource Center), and used to isolate the *myb71-1* mutant.

To growth plants for transformation and seeds production, seeds were soaked with sterile water and kept at 4 °C in darkness for 2–3 days, then sown into the soil pots directly and kept in a growth room. The pots were covered with plastic covers for 3–4 days to facilitate generation. The growth room was set with a cycle of 16 h for light with light density at approximately 125 µmol m^−2^ s^−1^ and 8h for darkness, the temperature in the growth room was set at 22 °C.

### 4.2. ABA Treatment, RNA Isolation and RT-PCR

To examine the response of *MYB71* (*At3g24310*), *MYB79* (*At4g13480*) and *MYB121* (*At3g30210*) to ABA, 10-day-old Col wild type seedlings grown in 1/2 MS plates were collected and treated with 50 μM ABA solution by shaking in darkness for 4 h, and the seedlings treated with solvent methanol were used as a control. The seedlings were then frozen in liquid nitrogen, and use for RNA isolation as described previously [18,35,62,63].

To examine the expression levels of *MYB71* in the transgenic plants, 10-day-old seedlings of the T3 homozygous transgenic plants grown in 1/2 MS plates were collected, frozen in liquid nitrogen and used for RNA isolation.

Total RNA was isolated by using an EasyPure PlantRNA Kit (TransGen Biotech, Beijing, China) according to the manufacturer’s instruction, DNA removal was conducted by treat the RNA isolated with DNase I for 15 min, and cDNA was synthesized by using an EasyScript First-strand DNA Synthesis Super Mix kit (TransGen Biotech, Beijing, China). If brief, 2 μg RNA was mixed with Reaction Mix, oligo(dT)_18_, Enzyme Mix and Rnase-free water, incubated at 42 °C for 30 min and then 85 °C for 5 s.

The cDNA was used as templates for RT-PCR analysis, 28 cycles were used for amplifying *MYB71*, *MYB79* and *MYB121*, and 24 for *ACT2* control with an annealing temperature of 56 °C. The primers used for RT-PCR analysis of *MYB71* are 5′-CAACATATGATGAGTTTGTGGGGAGGGATG-3′ and 5′-CAAGAGCTCTTAACAGAAGGGAATGACCATGTTC-3′, and for *MYB79* are 5′-CAACATATGATGGTGGAAGAAGTTTGGA-3′ and 5′-CAAGAGCTCTTAACAAAATGGAATCACC-3′, and for *MYB121* are 5′-CAACATATGATGCTTGATTGGGGAGTTCAAGGTC-3′ and 5′-CAAGAGCTCTCAAAAAATATAGCCTCCCATGTAAACTCCAG-3′. The primers for *ACT2* has been descried previously [64].

### 4.3. Constructs

To generate constructs for transcription activity assays, the full length ORF sequences of *MYB71*, *MYB79* and *MYB121* were amplified, respectively by RT-PCR, and used to the generation of *GD-MYB71*, *GD-MYB79* and *GD-MYB121* by cloning into the N-terminal GD tag-containing *pUC19* vector under the control of the *CaMV* double *35S* promoter [64,65]. The Gal4 DNA binding domain control construct *GD* and the *Gal4:GUS* reporter construct used for protoplast transfection were as described previously [66,67].

To generated constructs for the subcellular localization assay, the GD tag in the *GD-MYB71*, *GD-MYB79* and *GD-MYB121* constructs were replaced with a GFP tag to generated *GFP-MYB71*, *GFP-MYB79* and *GFP-MYB121* constructs respectively. The *NLS-RFP* nuclear indicator was as described previously [68].

To generate *35S:MYB71* construct for plant transformation, the GD tag in the *GD-MYB71* construct was replaced with a HA tag. To generate the *35S:MYB71-GR* construct for plant transformation, the full-length ORF of *MYB71* without stop condon was PCR amplified and cloned into the N-terminal HA tag- and C-terminal GR tag-containing *pUC19* vector. The *35S:MYB71* and *35S:MYB71-GR* constructs generated were then digested with corresponding restriction enzymes and cloned into the binary vector *pPZP211* [69].

To generate CRISPR/Cas9 genome editing construct for *MYB71*, the exon sequences of *MYB71* were used to identify target sequences on CRISPRscan (www.crisprscan.org, accessed on 11 January 2019), and then the identified target sequences were evaluated on Cas-OFFinder (www.rgenome.net/cas-offinder/, accessed on 11 January 2019). Two specific overlapped target sequences, i.e., 5′-GGGGAGGGATGGGAGGAGGA(TGG)-3′ and 5′-GGATGGGGAATGGTAGAAGA(AGG)-3′ were selected and cloned into the *pHEE401E* vector following the procedure as described previously [70]. The primers used to generate *MYB71* gene editing CRISPR/Cas9 construct were DT1-BsF, 5′-ATATATGGTCTCGATTGGGGAGGGATGGGAGGAGGAGTT-3′ and DT1-F0, 5′-TGGGGAGGGATGGGAGGAGGAGTTTTAGAGCTAGAAATAGC-3′, DT2-R0, 5′-AACTCTTCTACCATTCCCCATCCAATCTCTTAGTCGACTCTAC-3′ and DT2-BsR, 5′-ATTATTGGTCTCGAAACTCTTCTACCATTCCCCATCC-3′. The U626-IDF and U629-IDR primers used for colony PCR and sequencing the CRISPR/Cas9 construct generated have been described previously [70].

### 4.4. Plant Transformation, Transgenic Plant Selection and Cas9-Free Mutant Isolation

The Col wild type plants about 5-week-old with a few flowers in the main inflorescence were transformed by the floral dip method [71]. The plants were transformed via *GV3101* agrobacterium cells with the *35S:MYB71*, *35S:MYB71-GR* and the CRISPR/Cas9 constructs to obtain overexpression transgenic plants and gene edited mutants for *MYB71*, respectively. T1 seeds were collected from transgenic plants selected.

To select transgenic plants, the T1 seeds collected were sown on the plates with proper antibiotic. For overexpression transgenic plants selection, T1 seeds were sown on 1/2 MS plates containing 100 μg/mL Carbenicillin and 50 μg/mL Kanamycin. For gene editing transgenic plants selection, T1 seeds were sown on 1/2 MS plates containing 30 μg/mL Hygromycin and 100 μg/mL Carbenicillin.

To identify *35S:MYB71* and *35S:MYB71-GR* homozugous transgenic plants, T2 seeds from T1 transgenic plants were sown on 1/2 MS plates with 25 μg/mL Kanamycin to selected transgenic plants with a single T-DNA insertion (3:1 segregation of transgenic and wild type seedlings), and T3 seeds from T2 plants with a single T-DNA insertion were selected on 1/2 MS plates with 25 μg/mL Kanamycin to identify homozygous transgenic plants. The expression level of *MYB71* in the homozygous transgenic plants was examined by RT-PCR to identify overexpression transgenic plants.

To identify transgene-free gene edited mutants for *MYB71*, gene editing status of *MYB71* in T1 plants was examined by amplifying and sequencing the genomic sequence of *MYB71*. T2 seeds from gene edited T1 plants were sown in soil pots, and transgene-free T2 plants were identified by amplifying Cas9 fragment as described previously [36,72]. Gene editing status of *MYB71* in the transgene-free T2 plants was then examined to identify homozygous mutants.

### 4.5. DNA Isolation and PCR

To confirm the T1 *35S:MYB71* and *35S:MYB71-GR* transgenic plants obtained, DNA was isolated from leaves of T1 plants, a few days after the seedling were transferred into soil pots, by using DNA isolation buffer as described previously [66], and used for PCR analysis. The *HA* primer 5′-CCATGGGATACCCTTACGATG-3′ and the reverse primer used to amplify *MYB71* CDS were used to confirm the *35S:MYB71* and *35S:MYB71-GR* T1 transgenic plants.

To examine gene editing status of the T1 transgenic plants of the gene editing transgenic plants, and the T2 transgene-free plants, DNA was isolated from leaves of T1 plants and T2 plants a few days after the seedling were transferred into soil pots, and genome sequence of *MYB71* was PCR amplified, for 32 cycles with an annealing temperature of 56 °C by using primers 5′-AGGTTTCACAAGACAGAGAGAC-3′ and 5′-CAAGAGCTCTTAACAGAAGGGAATGACCATGTTC-3′ and sequenced.

### 4.6. Plasmid DNA Isolation, Protoplasts Isolation, Transfection, GFP Observation and GUS Activity Assays

The plasmids DNA including the reporter and effector constructs, the *GFP* and *NLS-RFP* subcellular localization constructs were isolated by using a GoldHi Endo Free Plasmid Maxi Kit (CWBIO, Beijing, China) from transformed *E. coli* cells, and DNA concentration was measured by using a NanoDrop (Thermo, Waltham, MA, USA) machine.

Rosette leaves from ~3 to 4 weeks old Col wild type plants were used to isolation protoplasts, and protoplasts were transfected by using a procedure described previously [64,73,74]. For transcription activity assays, the *GD*, *GD-MYB71*, *GD-MYB79* and *GD-MYB121* effects were co-trasfected, respectively, with the *Gal4:GUS* reporter construct plasmids DNA into the protoplasts isolated, and the transfected protoplasts were incubated at room temperature for 20–22 h under darkness. GUS activities were then assayed by using a Synergy TM HT fluorescence microplate reader (BioTEK, VT, USA).

For the subcellular localization assay, plasmids of *MYB71-GFP*, *MYB79-GFP* and *MYB121-GFP* were co-transfected respectively with *NLS-RFP*, and the transfected protoplasts were incubated at room temperature for 18–20 h under darkness. GFP and RFP fluorescence were then observed under a confocal microscope (Olympus, Tokyo, Japan).

### 4.7. ABA Response Assays

ABA inhibited seed germination and cotyledon greening were used to examine ABA response of the overexpression transgenic plants and gene edited mutants of *MYB71*, by using a procedure described previously [35,75,76,77]. Briefly, seeds of the Col wild type, the *35:MYB71* overexpression transgenic plants and *MYB71* mutants were sterilized and sowed on the 1/2 MS plates containing 1 µM ABA, or the solvent methanol as control. The plates were kept at 4 °C for 2 days in darkness and then transferred into the growth room. The numbers of seeds germinated were counted every 12 h, and photographs were taken 12 days after the transfer.

### 4.8. Transcriptome Analysis

*35S:MYB71-GR* seeds were sown on 1/2 MS plates and kept for 2 days at 4 °C in darkness. Then the plates were transferred into the growth room and grown for 10 days. The *35S:MYB71-GR* seedlings were then treated with 10 µM DEX or solvent only as control by shaking for 4 h. The seedlings were then frozen in liquid nitrogen, and send to the company (Beijing Genomics Institute, Shenzhen, China) for transcriptome sequencing and analysis. A potion of RNA isolated was returned by the company for result verification purpose. Genes upregulated at least 2 folds by DEX treatment were subject to functional categorization on TAIR Gene Ontology (GO) Annotation (https://www.arabidopsis.org/tools/bulk/go/index.jsp, accessed on 6 April 2021) under the term of biological process.

## 5. Conclusions

Our results in this research show that the *MYB71* is an ABA responsive gene, *MYB71* functions as a transcription activator, and it regulates plant ABA response in *Arabidopsis*.

## Data Availability

All data are presented in the manuscript and the Supplementary Material.

## Supplementary Materials

The following supporting information can be downloaded at: https://www.mdpi.com/article/10.3390/plants11101369/s1, Table S1: Genes upregulated in the *35S:MYB-GR* transgenic plants after DEX treatment.
